# The Role of Alexithymia and Impulsivity in Male Victims and Perpetrators of Intimate Partner Violence

**DOI:** 10.3390/bs13050402

**Published:** 2023-05-11

**Authors:** Stefania Mannarini, Federica Taccini, Alessandro Alberto Rossi

**Affiliations:** 1 Department of Philosophy, Sociology, Education, and Applied Psychology, Section of Applied Psychology, University of Padua, 35131 Padua, Italy; stefania.mannarini@unipd.it (S.M.); a.rossi@unipd.it (A.A.R.); 2 Centre for Intervention and Research on Family (CIRF)—Department of Philosophy, Sociology, Education, and Applied Psychology, Section of Applied Psychology, University of Padua, 35131 Padua, Italy; 3 Department of Developmental Psychology and Socialisation, University of Padua, 35131 Padua, Italy

**Keywords:** violence, intimate partner violence, alexithymia, impulsivity

## Abstract

(1) Background: Alexithymia and impulsivity appear to represent risk factors for violence perpetration, while mixed results are present with respect to victimization experience. In light of this, the purpose of this study was to compare the roles of both alexithymia and impulsivity among three different samples of men: men who experienced partner victimization (IPVV); male intimate partner violence perpetrators (IPVP); and men from the general population (CG). (2) Methods: Participants were recruited from specialized centers in Italy. A profile analysis was conducted. (3) Results: The results showed that IPVV presented alexithymia and impulsivity comparable to the CG. Furthermore, differences were found between victims and perpetrators in terms of impulsivity and alexithymia. The IPVP group had higher levels of both impulsivity and alexithymia in comparison to IPVV. Moreover, the perpetrators exhibited significantly higher levels of alexithymia compared to the CG. However, despite the medium Cohen’s d (*d* = 0.441) resulting from the analyses, IPVP’s level of impulsivity was not statistically different from the CG. (4) Conclusion: Alexithymia and impulsivity appear to play a key role in violent behaviors and should be the focus of psychological interventions with perpetrators.

## 1. Introduction

Intimate partner violence (IPV) refers to any conduct that causes physical, sexual, or psychological harm to individuals in a romantic relationship [1]. IPV perpetrated by men is highly prevalent throughout the world, with around 26% of women experiencing physical and/or sexual violence from a current or former male partner at least once in their lives since the age of 15 [2,3,4,5,6]. Regarding male victims, Kolbe and colleagues showed that the prevalence rates of domestic physical abuse against men ranged from around 3.4% to 20.3% [7]. In most cases, these men had also been violent toward their partners [7,8].

Alexithymia and impulsivity have been identified in the literature as risk factors for violence perpetration [9,10]. Alexithymia corresponds to difficulties in identifying, naming, cognitive processing, and regulating emotions. Moreover, people with clinical levels of alexithymia can present an externally oriented style of thinking and struggle to discriminate their subjective feelings from bodily ones of emotional arousal [11]. The literature shows that higher levels of alexithymia are linked to aggressive behaviors [12,13]. In this regard, Strickland and colleagues investigated the alexithymia levels of three different groups of male participants: 79 general violent perpetrators, 31 IPV perpetrators (IPVP), and 80 men for the control group—using the Toronto Alexithymia Scale-20 (TAS-20), which is the most famous self-report of alexithymia [12]. The results showed that general violent perpetrators and IPVP reported statistically significant higher levels of alexithymia compared to the general population in total TAS-20 scores and in two of three subscales of this self-report (difficulty identifying and describing emotions but not externally oriented thinking) [12]. Consequently, aggression may represent a strategy that people with alexithymia use to regulate emotions they cannot express verbally [12,14].

Mixed results are present in IPVV alexithymia levels [15,16,17,18]. In fact, previous research showed the presence of statistically significant higher levels of alexithymia among IPVV compared to individuals in the general population and that alexithymia appeared to play a role in the development of posttraumatic symptoms [18]. However, other research contradicts these results, showing no statistically significant differences in alexithymia levels between these groups [9]. Despite these results, high levels of alexithymia appear to be present in people with antisocial personality disorder [19], in violent forensic psychiatric outpatients [14], in abusive partners [9,12], and in adolescent perpetrators [20]. Additionally, alexithymia is associated with some intrapersonal problems, such as increased physiological arousal [21] and impulsivity [22,23].

Impulsivity refers to the tendency to display behavior that is not well thought through or reflected or that does not consider the consequences involved [24]. Stanford and colleagues suggested a three-factor model of impulsivity: (1) Cognitive Impulsiveness—based on quick choices and decisions; (2) Motor Impulsiveness—based on actions and behaviors conducted without thinking things through; and (3) Non-Planning Impulsiveness—which is based on a lower tendency to make plans ahead [25]. Impulsive behaviors seem to be present in different psychiatric disorders, such as depression, bipolar disorder, borderline personality disorder, antisocial personality disorder, attention deficit/hyperactivity disorder (ADHD), as well as suicide attempts and they appear to be present in criminal perpetrators as well [25,26,27,28]. Moreover, impulsivity is related to aggressive behaviors e.g., [26,27,29,30]), which can be directed toward the self (self-harm) or against others. In this regard, several studies have investigated impulsivity in perpetrators e.g., [29,31,32,33,34,35]). Specifically, the study by Tharp and colleagues with 121 IPVP with alcohol use disorder showed that impulsiveness predicted sexual and psychological violence [33]. Furthermore, the study by Stuart and Holtzworth-Munroe showed that IPVP had statistically significant higher levels of impulsiveness than non-violent men [35]. However, only a few studies have investigated the role of impulsivity in victims of violence e.g., [36,37,38]), and the results in this regard are mixed. Some studies indicate that victims exhibit levels of impulsivity similar to those who have not experienced violence [36]. Conversely, other research suggests that impulsivity, in combination with substance abuse, can increase the likelihood of experiencing further sexual assault [38].

Considering the relevance that both impulsivity and alexithymia appear to have in the victimization phenomenon, this study aims to use a profile analysis to evaluate and compare the contribution of alexithymia and impulsivity to IPV perpetration and victimization in men. Based on previous research, we predict that IPVP will exhibit significantly higher levels of alexithymia and impulsivity compared to the general population and the IPVV group. In addition, we hypothesize that IPVV will demonstrate levels of alexithymia and impulsivity similar to those of the general population.

## 2. Materials and Methods

### 2.1. Procedure

Each participant was recruited individually: a research survey assessing demographic information and psychological variables with self-report questionnaires was administered in person to each participant.

In line with previous research [9], male IPVP and male IPVV were recruited from specialized centers for the treatment of perpetrators of IPV and victims, respectively. The survey was administered within 2 to 6 months of their arrival at the centers. All the centers were in the north of Italy. Lastly, according to previous studies [9], a control group (CG) was randomly enrolled from the general population of Italy through personal invitations and advertisements in universities, libraries, supermarkets, cafes, etc.

Inclusion criteria for the overall sample (general inclusion criteria) consisted of the following: being a native Italian speaker, being over 18 years old, being in a relationship or having ended the relationship at least three months before the assessment, and having the ability to complete the assessment procedure. Whereas inclusion criteria for both the IPVP and the IPVV group were applied (specific inclusion criteria). Men should have exclusively acted (IPVP group) or suffered (IPVV group) at least one episode of IPV within the last 6–18 months. Men who had both suffered from and acted on IPV were excluded from the study. On the contrary, men from the CG should never have acted or been subjected to any kind of IPV.

### 2.2. Sample Size Calculation

The required sample size for testing the hypothesis of the present study was computed a priori. The G*Power software was used [39]. First, considering the main analysis of this research (see Profile Analysis), the multivariate analysis of variance (MANOVA, global effect) family of statistics was chosen. Second, a single independent variable with three levels was specified, i.e., the three groups of men. Third, four different variables (dependent variables) were specified—i.e., four response variables (see the ‘instruments’ section) [9,40,41,42]. Fourth, the a priori effect size statistic was set at small values [43,44]: Pillai’s trace (V) was set to 0.25—resulting in a small effect size: f^2^(V) = 0.111 [43,45]. Fifth, the Type I error (α) was set at 0.05 (two-sided), and the power (1—β) was set at 0.80 [43]. The results showed that there is more than 85% chance of correctly rejecting the null hypothesis of no significant effect of the model with an overall sample of 63 participants—21 participants per group.

### 2.3. Participants

An initial sample of 90 men was contacted. However, 14 men did not complete the procedure: 7 men IPVP, 3 men IPVV, and one man from the CG.

Thus, the final sample was composed of 76 participants. Men in IPVP were 25 and aged from 30 to 68 years (mean = 46.88, SD = 9.58). Forty percent were separated/divorced; 32% were married; 20% were in a relationship; and 8% were single. Men in the CG were 30 and aged from 27 to 65 years (mean = 45.30, SD = 12.70). 43.3% were married; 23.4% were separated/divorced; 20% were single; 13.3% were in a relationship; and 8% were single. Men in IPVV were 21 and aged from 24 to 54 years (mean = 41.24, SD = 8.53): 38.1% were separated/divorced; 28.6% were single; 23.8% were in a relationship; and 9.5% were married.

### 2.4. Measures

The demographic and clinical information form was used to collect: age, kind of interpersonal violence (acted or suffered), and actual relationship status.

#### 2.4.1. Toronto Alexithymia Scale 20 (TAS-20)

The TAS-20 [46] is one of the most widely used self-report questionnaires for the assessment of alexithymia. The TAS-20 was used in several contexts related to IPV—for example, with men/women who acted in IPV as well as with victims [15,47]. The TAS-20 evaluates the three main facets of alexithymia [11]: (A) difficulties in identifying feelings (DIF); (B) difficulties in describing feelings to other people (DDF); and (C) externally oriented thinking (EOT). It consists of 20 items on a 5-point Likert-type scale, with higher scores reflecting higher levels of the measured facet. In this study, the Italian version of the TAS-20 was used, which provided good internal consistency. Total scores: Cronbach’s Alpha = 0.858; DIF: Cronbach’s Alpha = 0.860; DDF: Cronbach’s Alpha = 0.802; and EOT: Cronbach’s Alpha = 0.601.

#### 2.4.2. Barratt Impulsiveness Scale (BIS-11)

The BIS-11 [25,48,49] is one of the most widely used self-report questionnaires for the assessment of impulsivity. The BIS-11 was used in settings associated with IPV—such as with men/women who acted IPV as well as in victims [32,33,34]. The BIS-11 evaluates the three main facets of impulsivity: (A) attentional impulsiveness, (B) motor impulsiveness, and (C) no-planning impulsiveness. However, a general total score was strongly assumed [49]. It consists of 30 items on a 4-point Likert-type scale, with higher scores reflecting higher levels of impulsivity. In this study, the Italian version of the BIS-11 was used, and it provided good internal consistency. Total score: Cronbach’s Alpha = 0.702.

### 2.5. Statistical Analyses

Data analysis was performed using JASP and R software and the following packages: ‘esvis’ [50], ‘ggplot2′ [51], ‘psych’ [52], and ‘tidyverse’ [53].

First, preliminary analyses [42] were performed: normality, linearity, multicollinearity, and homogeneity of covariance matrices.

Second, a profile analysis (PA) was performed [54]. PA is a specific multivariate approach to testing mean differences (MANOVA family of statistics) that allows to quantify the extent to which groups of individuals (three groups: independent variables) revealed different profiles on variables implied in IPV (four constructs: dependent variables)—computing the amount of difference among profiles [40,55,56,57,58]. According to the guidelines, before performing PA, all dependent variables were rescaled into z-scores [40,42]. PA provides three specific statistics: (I) parallelism; (II) level equality; and (III) flatness [41,42,58,59]. (A) Parallelism assesses whether two profiles are analogous and symmetrical (parallel) among different groups (namely, between-subject general statistics). (B) Level equality refers to the degree of similarity (equal means scores) of all of the dependent variables across groups—a general between-subject statistic. To test the level of equality, several focused comparisons between groups were performed [60]. (C) Flatness determines (within each profile) whether each variable yielded a similar response to the other variables—a general within-subjects statistic [42]. To test flatness, several focused repeated measures comparisons were also performed—separately—for each group (within-group effect).

Bonferroni’s correction was applied. Partial eta-square (η^2^_p_) and Cohen’s d were used to quantify the difference in multiple and pairwise comparisons, respectively, with the following benchmarks: small (η^2^_p_: 0.011 to 0.059; d: 0.20 to 0.49), moderate (η^2^_p_: 0.060 to 0.139; d: 0.50 to 0.79), and large (η^2^_p_ > 0.140; d > 0.80) [42,43]. In addition, it is important to note that due to the small sample size, differences in means between groups are not likely to be statistically significant; therefore, the results will be interpreted using effect sizes.

## 3. Results

### 3.1. Preliminary Analyses: Controlling Model Assumptions

Preliminary analyses revealed that each psychological variable was normally distributed (univariate normality) and that the bivariate relationships among each pair of dependent variables were almost linear (a scatter matrix that revealed no curvilinear relationships). At the same time, Pearson’s bivariate correlation coefficients showed that the bivariate relationships did not exceed the critical cut-off of |0.80|. Moreover, tolerance and variance inflation factor (VIF) statistics showed the absence of multicollinearity. The results are reported in Table 1.

Finally, the Box’s M test (homogeneity of variance-covariance matrices) resulted in being statistically significant (M = 35.759, F = 1.642, *p* < 0.035)—due to its overpower related to groups with equal size, as in this case [9,54]. Thus, the PA was carried out [42,60].

### 3.2. Preliminary Analyses: Assessment of General Alexithymia Levels among Groups

As an additional preliminary analysis, general levels of alexithymia were investigated among the three groups of individuals. ANOVA revealed statistically significant differences between the three groups: F = 6.839, *p* = 0.001, and η^2^p = 0.158 (large effect size). Furthermore, specific between-group comparisons were investigated. A statistically significant difference was found between IPVV (M = 40.24, SD = 11.238) and IPVP (M = 53.72, SD = 12.068): t = −3.606, *p* = 0.002, *d* = −1.07 (large effect size). Moreover, a statistically significant difference was found between the IPVP (M = 53.72, SD = 12.068) and the CG (M = 45.17, SD = 13.926): t = 2.501, *p* = 0.044, *d* = 0.68 (moderate effect size). Lastly, no statistically significant differences were found between IPVV (M = 40.24, SD = 11.238) and CG (M = 45.17, SD = 13.926): t = −1.372, *p* = 0.523, *d* = −0.39 (small effect size).

### 3.3. Profile Analysis: Parallelism

**Figure 1:**
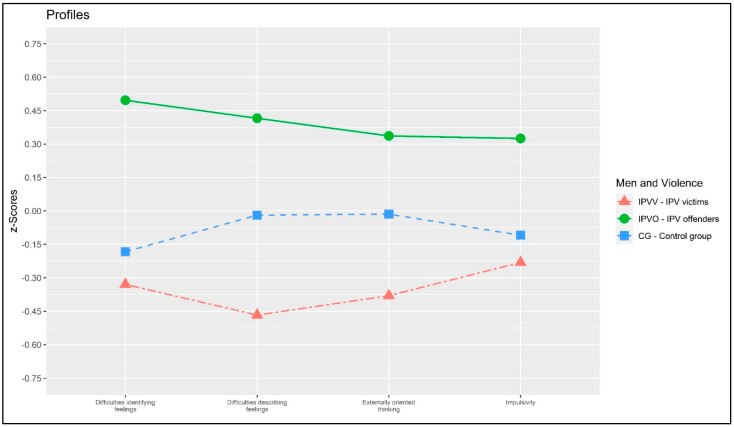
Plot of the profile analysis.

A statistically significant effect of the group of participants (IPVV vs. IPVP vs. CG) was found on variables related to IPV, revealing the presence of parallelism among profiles: Pillai’s V = 0.052, F = 0.637, *p* = 0.700, η^2^p = 0.026 (small effect size). Figure 1 represents the presence of parallelism.

### 3.4. Profile Analysis: Level Equality—Between-Group Differences

A statistically significant between-group effect was found: F = 6.552, *p* = 0.002. This result confirmed that the three groups were, on average, different.

**Figure 2:**
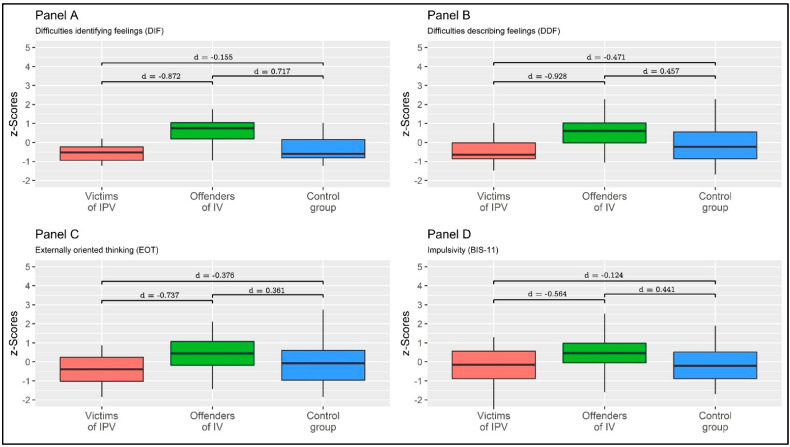
Between-group means comparison. Values are expressed as z-scores. Note: Independent samples of Cohen’s *d*s were reported.

The multivariate pairwise focused contrast between IPVV and IPVP showed a statistically significant multivariate effect: Pillai’s V = 0.268, F = 3.747, *p* = 0.011, η^2^p = 0.268 (large effect size). Moreover, multivariate pairwise focused contrast between the IPVV and the CG showed a non-statistically significant multivariate effect: Pillai’s V = 0.077, F = 0.959, *p* = 0.439, η^2^p = 0.077 (small effect size). Lastly, a multivariate pairwise focused contrast between IPVP and the CG showed a non-statistically significant multivariate effect: Pillai’s V = 0.125, F = 1.792, *p* = 0.145, η^2^p = 0.125 (moderate effect size). Specific between-group comparisons were reported in Table 2 and Figure 2.

### 3.5. Profile Analysis: Flatness—Within-Group Differences

A non-statistically significant within-group effect was found: F = 0.498, *p* = 0.783, η^2^p = 0.013 (small effect size).

**Figure 3:**
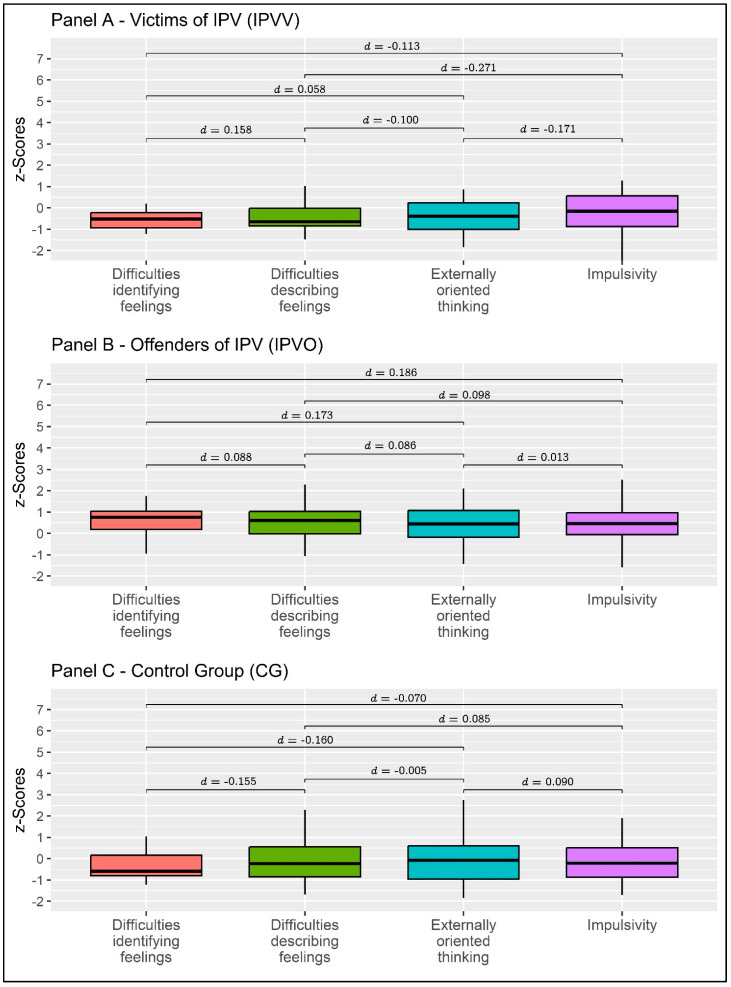
Between-group means comparison. Values are expressed as z-scores. Note: Dependent samples of Cohen’s *d*s were reported.

Considering IPVV individuals, a non-statistically significant multivariate effect was found: Pillai’s V = 0.063, F = 0.401, *p* = 0.754, η^2^p = 0.063 (moderate effect size). Considering IPVP individuals, a non-statistically significant multivariate effect was found: Pillai’s V = 0.110, F = 905, *p* = 0.455, η^2^p = 0.110 (moderate effect size). Lastly, considering individuals from the CG, a non-statistically significant multivariate effect was found: Pillai’s V = 0.036, F = 0.341, *p* = 0.769, η^2^p = 0.036 (small effect size). Detailed results—bivariate comparisons—are reported in Table 3 and Figure 3.

## 4. Discussion

The objective of the present study was to investigate alexithymia and impulsivity in victims and perpetrators of IPV. To reach this objective, a profile analysis was conducted among three different samples of men: men who experienced partner victimization, male perpetrators, and men from the general population.

First, the results showed a statistically significant correlation between impulsivity and alexithymia. Consequently, people who struggle to identify and label their own and others’ feelings and emotions can use impulsive behaviors (such as impulsive aggression) to cope with these difficulties [12,14,26,29]. This is in line with previous literature on the matter, which shows that the ability of a person to identify and, then, categorize negative emotional experiences moderates the relationship between anger and aggressive behaviors [61].

Furthermore, the results confirmed our hypotheses, showing differences between IPVV and IPVP. Specifically, perpetrators had higher levels of impulsivity and alexithymia compared to victims. This is in line with previous literature showing that both impulsivity and alexithymia appear to be related to higher levels of aggression [27,29,62]. In this regard, the study by Garofalo and colleagues showed that emotion dysregulation and impulsivity mediated the relationship between alexithymia and aggression. Consequently, a person’s ability to regulate emotions and control impulsive behaviors seems to play a key role in the relationship between alexithymic difficulties and aggressive behavior [62]. Furthermore, alexithymia represents a potential risk factor for reoffending in intimate relationships and for dropping out of psychotherapeutic support [63]. Therefore, this result contributes to highlighting the importance of considering the role of alexithymia and impulsivity in understanding IPV and developing effective interventions for IPVP. The findings suggest that addressing these psychological constructs in the early stages of the psychological intervention with IPVP can play a key role in reducing aggression, improving psychotherapeutic outcomes, and reducing drop-out rates.

In addition, in line with our hypothesis, the between-group comparison showed statistically significant differences between IPVP and CG in alexithymia levels. Specifically, the perpetrators had higher levels of difficulty describing and identifying feelings and emotions. This aligns with previous research indicating that individuals with alexithymic traits can struggle with empathizing with others and understanding their feelings [9,64]. As a result, this may cause difficulties in their ability to control their behaviors, leading to more violent behaviors toward their intimate partners [47].

Several studies have pointed out that alexithymia is related to difficulties in interpersonal relationships, such as empathizing with others’ emotions [65,66], which, in this case, may contribute to further violence. It is important to note that IPV is often characterized by a cycle of violence, in which violence and abuse escalate over time [67]. This implies that the more perpetrators struggle to understand their partners’ emotions and express their own, the more likely they are to escalate their violent behaviors toward their partners. These findings highlight the importance of addressing alexithymic difficulties in the early stages of psychological interventions with IPVP. By doing so, it may be possible to prevent the cycle of violence from escalating and reduce the risk of reoffending. Contrary to expectations, the CG did not differ statistically significantly from the IPVP in the impulsivity level. This result may reflect the small sample size. In fact, the analysis resulted in a medium Cohen’s d value (*d* = 0.441), indicating a relationship between impulsivity and aggression. This suggests that individuals who struggle with mentalizing affective states may be more likely to use maladaptive strategies, such as aggressive behaviors [68,69].

In conclusion, our hypotheses were supported by the findings and are consistent with some of the literature (e.g., [9]), indicating that IPVV did not exhibit alexithymic difficulties. Additionally, their impulsivity levels were comparable to those of the CG. This outcome emphasizes that these psychological factors may not contribute to the experience of victimization; hence, they should not be the primary focus of interventions with victims of violence. Instead, interventions should primarily target perpetrators who have been found to have higher levels of alexithymia and impulsivity.

### 4.1. Implications for Practice

These results contribute to the suggestion that alexithymia should be evaluated and addressed in rehabilitation programs for violence perpetrators. In fact, the literature shows that perpetrators could benefit from training in emotional identification and labeling skills and anger management techniques [12,63]. Additionally, group therapy could be implemented in these cases, as patients with higher levels of alexithymia could perceive it as less demanding from an emotional and relational point of view [70,71]. Moreover, according to the results, impulsivity should also be addressed in clinical interventions with IPVP. For example, cognitive-behavioral therapy techniques such as self-control training, problem-solving skills, and coping skills appear to be one of the focuses of intervention [72,73].

In summary, psychological interventions for IPV perpetrators to be successful should target the perpetrators’ lack of ability to identify and express emotions and control their impulsive behaviors.

### 4.2. Limitations

Some limitations of this study must be acknowledged. First, a cross-sectional study design was implemented in this study. Although cross-sectional studies are widely used, they suffer from a number of flaws, such as simultaneous measurement of exposure and outcome. This often makes causality difficult to pin down. [74]. Second, the sample size was small, even though it was still sufficient for the statistical analysis conducted in the present manuscript.

In future studies, it would be beneficial to increase the sample size and examine the role of emotion regulation, in line with previous research. Additionally, it is recommended to conduct studies on a national level and include cross-cultural comparisons.

## 5. Conclusions

The present study aimed to investigate alexithymia and impulsivity in victims and perpetrators of IPV. A profile analysis was conducted. The results showed that IPVV presented levels of alexithymia and impulsivity comparable to the general population. In contrast, IPVP showed difficulties in identifying and describing emotions and higher levels of impulsivity. Therefore, these two psychological constructs may play a key role in violent behaviors and should be targeted in the clinical setting.

## Figures and Tables

**Table 1:** Descriptive statistics of questionnaires (row scores) and correlations between variables.

		Descriptive Statistics				Correlations					Collinearity	
		M	SD	Sk	K	1	2	3	4	5	T	VIF
1	TAS-20	46.62	13.576	0.391	−0.696	-					-	-
2	DIF	15.66	7.051	0.698	−0.311	0.896 ***	-				0.407	2.460
3	DDF	13.09	4.781	0.399	−0.546	0.856 ***	0.723 ***	-			0.462	2.164
4	EOT	17.87	4.787	0.185	−0.338	0.681 ***	0.347 **	0.363 **	-		0.847	1.181
5	BIS-11	59.53	9.700	−0.030	−0.341	0.530 ***	0.548 ***	0.433 ***	0.264 *	-	0.692	1.445

Note: * *p* < 0.050; ** *p* < 0.010; *** *p* < 0.001. M = mean; SD = standard deviation; Sk = skewness; K = kurtosis; T = tolerance; VIF = Variance Inflaction Factor. TAS-20 = TAS-20 Total score; DIF = difficulties in identifying feelings; DDF = difficulties in describing feelings to other people; EOT = externally oriented thinking; BIS-11 = Barratt Impulsiveness Scale-11.

**Table 2:** Between-group comparisons.

		IPVV		IPVP		CG		IPVV vs. IPVP		IPVP vs. CG		IPVV vs. CG	
		M	SD	M	SD	M	SD	*t*	*d*	*t*	*d*	*t*	*d*
1	DIF	13.33	6.621	19.16	5.640	14.37	7.472	−2.946 *	−0.872	2.649 *	0.717	−0.544	−0.155
2	DDF	10.86	3.229	15.08	4.349	13.00	5.408	−3.136 **	−0.928	1.689 *	0.457	−1.656	−0.471
3	EOT	16.05	3.827	19.48	4.473	17.80	5.288	−2.489 *	−0.737	1.332	0.361	−1.322	−0.376
4	BIS-11	57.29	10.061	62.68	10.007	58.47	8.792	−1.907	−0.564	1.628	0.441	−0.434	−0.124

Note: * *p_bonf_* < 0.050; ** *p_bonf_* < 0.010; IPVV = IPV victims; IPVP = IPV perpetrators; CG = Control group; M = mean; SD = standard deviation; *t* = independent sample *t*-test; *d* = Cohen’s d (effect size); DIF = difficulties in identifying feelings; DDF = difficulties in describing feelings to other people; EOT = externally oriented thinking; BIS-11 = Barratt Impulsiveness Scale-11.

**Table 3:** Within-group comparisons.

		IPVV		IPVP		CG	
		*t*	*d*	*t*	*d*	*t*	*d*
DIF	DDF	0.595	0.158	0.447	0.088	−0.767	−0.155
	EOT	0.219	0.058	0.884	0.173	−0.790	−0.160
	BIS-11	−0.426	−0.113	0.948	0.186	−0.346	−0.070
DDF	EOT	−0.376	−0.100	0.437	0.086	−0.023	−0.005
	BIS-11	−1.020	−0.271	0.501	0.098	0.421	0.085
EOT	BIS-11	−0.644	−0.171	0.064	0.013	0.444	0.090

Note: IPVV = IPV victims; IPVP = IPV perpetrators; CG = Control group; M = mean; SD = standard deviation; *t* = dependent sample *t*-test; *d* = Cohen’s d for paired sample (effect size); DIF = difficulties in identifying feelings; DDF = difficulties in describing feelings to other people; EOT = externally oriented thinking; BIS-11 = Barratt Impulsiveness Scale-11.

## Data Availability

Due to privacy restrictions, the dataset used in the current study is available from the corresponding author on reasonable request.
